# Ginsenoside Rh_2_ Alleviates LPS-Induced Inflammatory Responses by Binding to TLR_4_/MD-2 and Blocking TLR_4_ Dimerization

**DOI:** 10.3390/ijms25179546

**Published:** 2024-09-02

**Authors:** Shujuan Pan, Luyuan Peng, Qion Yi, Weijin Qi, Hui Yang, Hongying Wang, Lu Wang

**Affiliations:** 1 School of Pharmaceutical Sciences, Guizhou University, Guiyang 550025, China; 18216620949@163.com (S.P.); 13002275214@163.com (H.W.); 2 Engineering Research Center of the Utilization for Characteristic Bio-Pharma Ceutical Resources in Southwest, Ministry of Education, Guizhou University, Guiyang 550025, China; yiqiong19870328@126.com (Q.Y.); 18286364283@163.com (W.Q.); m15735011560@163.com (H.Y.); 3 College of Veterinary Medicine, Jilin University, Changchun 130062, China; pengly1992@163.com

**Keywords:** G-Rh_2_, inflammation, TLR_4_ signaling pathway, target, TLR_4_/MD-2

## Abstract

Lipopolysaccharide (LPS) triggers a severe systemic inflammatory reaction in mammals, with the dimerization of TLR_4_/MD-2 upon LPS stimulation serving as the pivotal mechanism in the transmission of inflammatory signals. Ginsenoside Rh_2_ (G-Rh_2_), one of the active constituents of red ginseng, exerts potent anti-inflammatory activity. However, whether G-Rh_2_ can block the TLR_4_ dimerization to exert anti-inflammatory effects remains unclear. Here, we first investigated the non-cytotoxic concentration of G-Rh_2_ on RAW 264.7 cells, and detected the releases of pro-inflammatory cytokines in LPS-treated RAW 264.7 cells, and then uncovered the mechanisms involved in the anti-inflammatory activity of G-Rh_2_ through flow cytometry, fluorescent membrane localization, Western blotting, co-immunoprecipitation (Co-IP), molecular docking and surface plasmon resonance (SPR) analysis in LPS-stimulated macrophages. Our results show that G-Rh_2_ stimulation markedly inhibited the secretion of LPS-induced interleukin-6 (IL-6), tumor necrosis factor-α (TNF-α) and nitric oxide (NO). Additionally, G-Rh_2_ blocked the binding of LPS with the membrane of RAW 264.7 cells through direct interaction with TLR_4_ and MD-2 proteins, leading to the disruption of the dimerization of TLR_4_ and MD-2, followed by suppression of the TLR_4_/NF-κB signaling pathway. Our results suggest that G-Rh_2_ acts as a new inhibitor of TLR_4_ dimerization and may serve as a promising therapeutic agent against inflammation.

## 1. Introduction

Toll-like receptor 4 (TLR_4_), a member of the toll-like receptor (TLR) family, is involved in innate immunity and mediates inflammatory responses by recognizing lipopolysaccharide (LPS) [1,2]. LPS-induced bimolecular TLR_4_/MD-2 dimerization is the basis of LPS-induced signal transduction. The lipid A component of *Escherichia coli* LPS features two phosphorylated glucosamines linked by a β (1–6) linkage and acylated by six lipid chains. Among these, five fatty acid chains are deeply embedded within a pocket, while the sixth chain extends to the surface of MD-2, engaging in hydrophobic interactions with the invariant phenylalanine residues of the adjacent TLR_4_*. Subsequently, the two phosphate groups on lipid A attach to the TLR_4_/MD-2 complex through electrostatic interactions with positively charged amino acids in TLR_4_, TLR_4_* and MD-2 [3]. The formation of the TLR_4_/MD-2/TLR_4_*/MD-2* heterodimer ultimately triggers the dimerization of the cytoplasmic domains, culminating in the recruitment of downstream adaptor proteins and the initiation of intracellular signaling cascades, ultimately leading to an immune response [4]. Studies have shown that TLR_4_ dimerization is crucial for activating downstream inflammatory signaling pathways, including the NF-κB pathway, which in turn promotes the secretion of TNF-α, IL-6 and IL-1β [5,6]. Several studies have demonstrated that a range of substances functioning as TLR_4_/MD-2 inhibitors can produce anti-inflammatory outcomes by blocking the dimerization of TLR_4_ with MD-2, such as total tanshinones, isoacteoside and ginsenoside Rb1 [7,8,9]. Therefore, blocking TLR_4_ dimerization has become a new strategy to inhibit inflammation.

G-Rh_2_, first isolated from red ginseng, is a steroidal saponin belonging to the protopanaxadiol type, and has various potent biological functions, including antitumor, anti-obesity, anti-inflammatory and antioxidant activities, preventing neurodegenerative diseases and so on [10,11,12,13,14]. In this study, we aimed to explore the anti-inflammatory effect of G-Rh_2_ by constructing an LPS-induced RAW 264.7 cell inflammatory model in vitro. Furthermore, we determined whether G-Rh_2_ blocked the TLR_4_/MD-2 dimerization to exert anti-inflammatory effects.

## 2. Results

### 2.1. Effect of G-Rh_2_ on the Levels of TNF-α, IL-6 and NO

**Figure 1:**
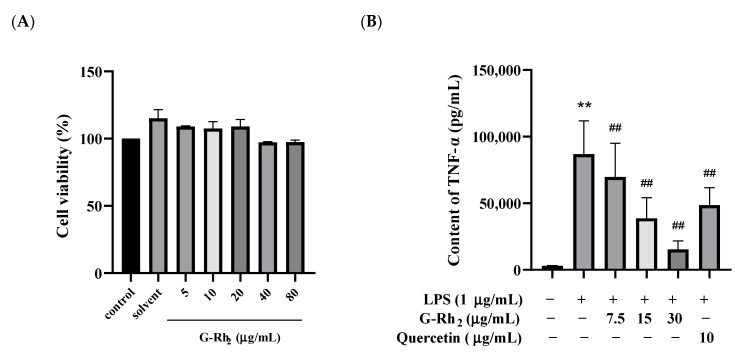

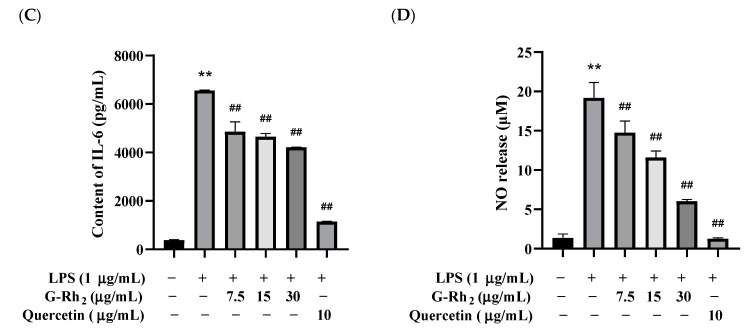
G-Rh_2_ inhibited LPS-induced inflammatory cytokine expression in RAW 264.7 cells in vitro. After treatment with different concentrations of G-Rh_2_, (**A**) the cell viability was measured by MTT. LPS was incubated with RAW 264.7 cells alone or together with G-Rh_2_ for 12 h, and the secretion levels of (**B**) TNF-α, (**C**) IL-6 and (**D**) NO were detected in the supernatant of the medium. The data are presented as mean values ± standard deviation (*n* = 3), based on a minimum of three separate experiments (**, *p* < 0.01, compared with the control group. ##, compared with the LPS group, *p* < 0.01).

To explore whether G-Rh_2_ exerted potential cytotoxicity action on RAW 264.7 cells, cell viability was detected by an MTT assay after incubation for 24 h in the absence or presence of different concentrations of G-Rh_2_. Our findings indicated that G-Rh_2_, at concentrations ranging from 0 to 80 μg/mL, did not notably impact cell viability in a dose-dependent manner (*p* > 0.05) (Figure 1A). 

Because TNF-α, IL-6 and NO are the main markers of inflammation, we analyzed the effects of G-Rh_2_ on TNF-α, IL-6 and NO secretion in LPS-induced RAW 264.7 cells in vitro. As shown in Figure 1B–D, TNF-α, IL-6 and NO secreted by RAW 264.7 cells were significantly increased under LPS stimulation (*p* < 0.01). Compared with the LPS group, the secretion of TNF-α, IL-6 and NO was significantly decreased by different concentrations of G-Rh_2_ (*p* < 0.01). Among these, the inhibitory effects of G-Rh_2_ (15 and 30 μg/mL) on TNF-α secretion were better than those of the positive drug (quercetin 10 μg/mL).

### 2.2. Effect of G-Rh_2_ on the Binding of FITC-LPS to the Cell Membrane

**Figure 2:**
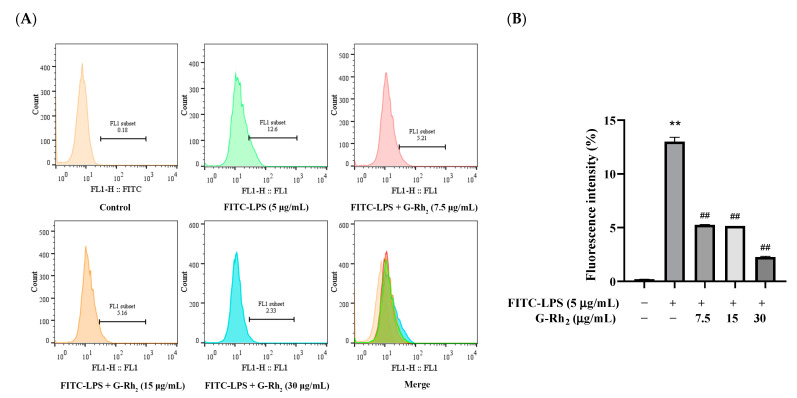
Effect of G-Rh_2_ on FITC-LPS binding to RAW 264.7 cells. LPS was incubated with RAW 264.7 cells alone or together with G-Rh_2_ for 6 h. Cells were collected and the mean fluorescence values of cells in each group were detected by flow cytometry. (**A**) The bound FITC-LPS with RAW 264.7 was examined by flow cytometry. (**B**) The fluorescence intensity was analyzed. The results are shown as means ± SD (*n* = 3) of at least three independent experiments (**, *p* < 0.01, compared with the mean fluorescence values of cells in the control group. ##, compared with the mean fluorescence values of cells in the LPS group, *p* < 0.01).

To investigate the effect of G-Rh_2_ on the binding of FITC-LPS to the cell membrane, firstly, we further detected the effect of G-Rh_2_ on the binding of FITC-LPS to RAW 264.7 cells by flow cytometry. As shown in Figure 2, the fluorescence intensity in the FITC-LPS stimulation group was significantly increased to 12.6% (*p* < 0.01). And compared with the FITC-LPS group, the fluorescence intensity of G-Rh_2_ groups were significantly decreased to 5.21%, 5.16% and 2.33%, respectively (*p* < 0.01), in a concentration-dependent manner.

**Figure 3:**
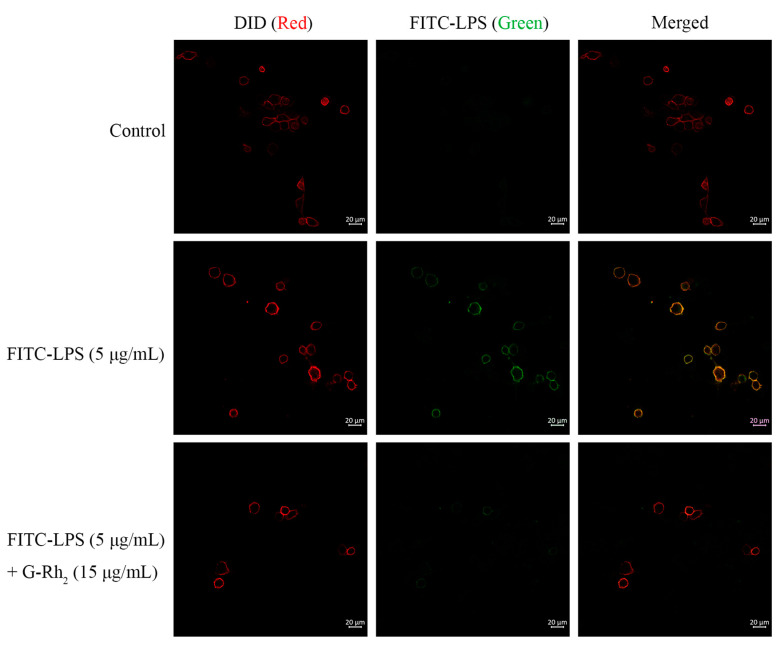
G-Rh_2_ blocks the localization of FITC-LPS on the cell membrane. Morphologically, the effect of G-Rh_2_ on the localization of FITC-LPS on the cell membrane was observed by CLSM. Protein on the cell membrane was observed as red fluorescence circles on the DID line; FITC-LPS with green fluorescence was bound to the membrane, and was observed as green circles on the cell membrane.

Secondly, we measured the fluorescence intensity of the cell membrane by CLSM, as shown in Figure 3. The cell membranes fluoresced strongly after FITC-LPS stimulation, whereas the G-Rh_2_ group fluoresced weakly.

### 2.3. Effect of G-Rh_2_ on the Activation of the TLR_4_/NF-κB Signaling Pathway

**Figure 4:**
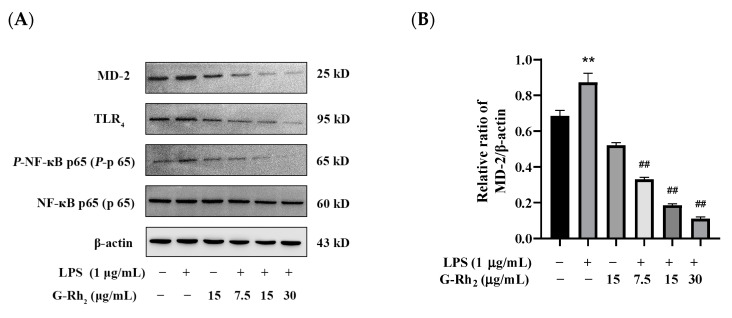

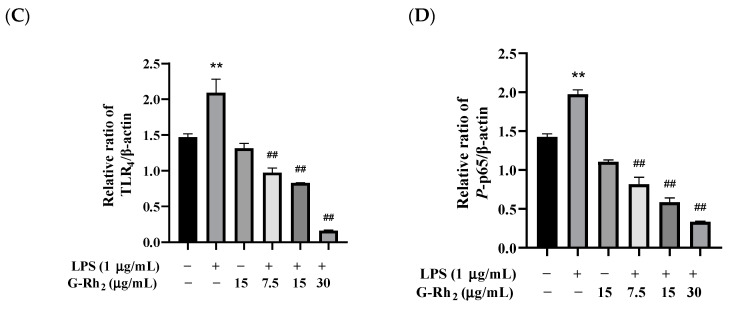
Effects of G-Rh_2_ on the expression of TLR_4_ signaling pathway proteins. (**A**–**D**) LPS was incubated with RAW 264.7 cells alone or together with G-Rh_2_ for 12 h. The effects of G-Rh_2_ on MD-2, TLR_4_ and *P*-NF-κB p65 were analyzed by Western blotting. The results are shown as means ± SD (*n* = 3) of at least three independent experiments (**, *p* < 0.01, compared with the control group. **##**, compared with the LPS group, *p* < 0.01).

To further confirm that G-Rh_2_ inhibits the production of pro-inflammatory factors by blocking the TLR_4_ signaling pathway, we examined the effect of G-Rh_2_ on the expression of key proteins of the TLR_4_ signaling pathway. As shown in Figure 4, G-Rh_2_ dose-dependently inhibited LPS-induced phosphorylation levels of NF-κB p65 and protein expression of TLR_4_ and MD-2 (*p* < 0.01). 

### 2.4. Effect of G-Rh_2_ on the Dimerization of TLR_4_ and MD-2

**Figure 5:**
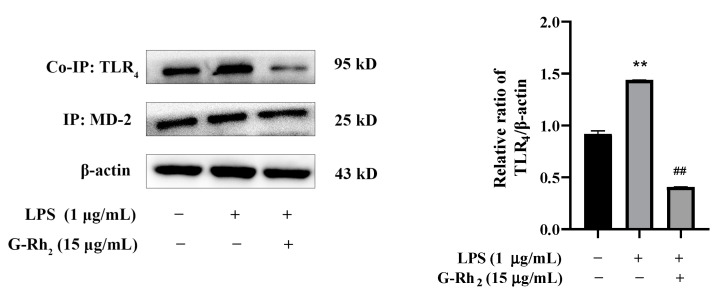
G-Rh_2_ inhibits the formation of the TLR_4_/MD-2 complex. A Co-IP assay detected the effect of G-Rh_2_ on the complex formation of TLR_4_ and MD-2. The results are shown as means ± SD (*n* = 3) of at least three independent experiments (**, *p* < 0.01, compared with the control group. ##, compared with the LPS group, *p* < 0.01).

Only the dimerization of TLR_4_ and MD-2 can trigger TLR_4_ intracellular signaling pathways and finally induce NF-κB into the nucleus [15]. Therefore, we further investigated the effect of G-Rh_2_ on LPS-induced dimerization of TLR_4_ and MD-2 by means of Co-IP. As shown in Figure 5, the co-precipitation of TLR_4_/MD-2 was significantly increased in the LPS group (*p* < 0.01), while G-Rh_2_ (15 μg/mL) treatment significantly inhibited LPS-induced TLR_4_/MD-2 complex formation (*p* < 0.01). The results indicated that G-Rh_2_ could prevent the transmission of LPS signals to cell membranes and inhibit the dimerization of TLR_4_ and MD-2. 

### 2.5. G-Rh_2_ Binds to TLR_4_/MD-2, Blocking the Formation of LPS-TLR_4_/MD-2 Complexes

**Figure 6:**
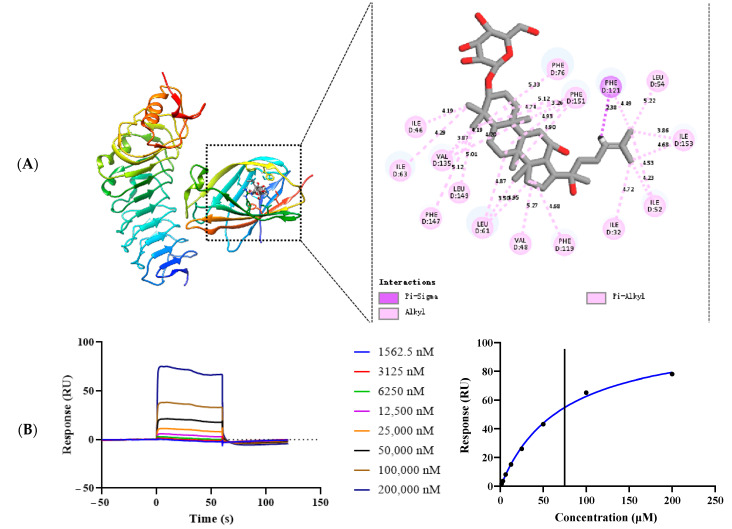
G-Rh_2_ targets TLR_4_/MD-2. (**A**) The amino acid interaction site between G-Rh_2_ and TLR_4_/MD-2 was investigated by molecular docking. (**B**) The interaction affinity between G-Rh_2_ and TLR_4_/MD-2 was studied with an SPR experiment.

To investigate the mechanism of G-Rh_2_ inhibiting the dimerization of TLR_4_ and MD-2, we performed molecular simulations of complexes formed by G-Rh_2_ and TLR_4_/MD-2 using molecular docking software (version 1.1.2). As shown in Figure 6A, G-Rh_2_ is mated into the hydrophobic pocket of TLR_4_/MD-2, which binds to TLR_4_/MD-2 and may present an amino acid binding site (ILE32, ILE46, VAL48, ILE52, LEU54, LEU61, ILE63, PHE76, PHE119, PHE121, VAL135, PHE147, LEU149, PHE151, ILE153).

Next, we evaluated the interaction between G-Rh_2_ and TLR_4_/MD-2 through SPR experiments; as shown in Figure 6B, G-Rh_2_ binds to TLR_4_/MD-2 protein in a dose-dependent manner and presents a “fast up, fast down” binding pattern. G-Rh_2_ and TLR_4_/MD-2 have a specific binding force and an affinity of KD = 71.20 µM. 

## 3. Discussion

G-Rh_2_, a protopanaxadiol saponin from ginseng, has been reported to exhibit anti-inflammatory and anticancer effects [16,17,18]. This provides strong support for us to further explore its anti-inflammatory mechanism. Therefore, the aim of this study was to verify the anti-inflammatory activity of G-Rh_2_, and to explore the underlying mechanisms in a cell inflammation model. Our results revealed that G-Rh_2_ could significantly decrease the secretion of TNF-α, IL-6 and NO in LPS-induced RAW 264.7 cells and indicated G-Rh_2_ exerted quite evident anti-inflammatory effects. Both TNF-α and IL-1β are reported to be NF-κB target genes, and the expression of these two target genes is significantly increased in LPS-stimulated macrophages [19]. Quercetin inhibits the NF-κB, Akt and JNK signaling pathway, thereby reducing the expression of TNF-α and IL-1β in LPS-stimulated RAW 264.7 cells [20]. We speculate that G-Rh_2_ could block the binding of LPS with the membrane of RAW 264.7 cells through direct interaction with TLR_4_ and MD-2 proteins, followed by suppression of the TLR_4_/MD-2 mediated downstream NF-κB signaling pathway, so as to better reduce the expression of TNF-α.

LPS, a macromolecular glycolipid, is unable to cross cell membranes by itself but must bind to membrane receptors to exert its biological effects [21,22]. To further explore the anti-inflammatory mechanism of G-Rh_2_ in LPS-induced RAW 264.7 cells, we next detected the effect of G-Rh_2_ on the binding of LPS to RAW 264.7 cells by flow cytometry, and further determined the effect of G-Rh_2_ on the binding of LPS to cell membranes with laser confocal technology. Flow cytometry showed that G-Rh_2_ could significantly inhibit the fluorescence intensity of FITC-LPS binding to the cells, indicating that G-Rh_2_ could significantly inhibit the binding of LPS to RAW 264.7 cells. Laser confocal analysis showed that G-Rh_2_ could significantly inhibit the fluorescence intensity of FITC-LPS binding to cell membranes. These results suggest that G-Rh_2_ may inhibit the binding of LPS to cell membranes to alleviate LPS-induced cell inflammation.

TLR_4_ is responsible for the recognition of LPS and then induces the activation of the NF-κB signaling pathway [23]. Therefore, we then explored the effects of G-Rh_2_ on the TLR_4_/NF-κB signaling pathway. Regarding the results, G-Rh_2_ significantly inhibited the protein expression of TLR_4_ and MD-2 and phosphorylation of NF-κB p65 in LPS-induced RAW 264.7 cells. These results indicated that G-Rh_2_ may block the combination of TLR_4_ and MD-2, and then inhibit TLR_4_/NF-κB signaling pathway activation. To further explore whether G-Rh_2_ interfered with the combination of TLR_4_ and MD-2, we explored whether G-Rh_2_ influences the LPS-induced dimerization of TLR_4_ and MD-2 by Co-IP. The findings demonstrated that the co-precipitation of TLR_4_/MD-2 exhibited a marked increase in the LPS group. Conversely, the administration of G-Rh_2_ markedly impeded the formation of the LPS-induced TLR_4_/MD-2 complex. 

According to the previous report, LPS-induced TLR_4_/MD-2 dimerization is the key to inflammation signaling transduction [24]. A previous study has reported that anthocyanins mostly fit into the hydrophobic pocket of MD-2 and bind to TLR_4_, which results in the inhibition of NF-κB activity to attenuate lipopolysaccharide-induced inflammation [25]. TTAK-242 inhibits the activation of the TLR_4_ signaling pathway by binding to the Cys747 amino acid site of TLR_4_’s intracellular TIR domain [26,27]. However, how G-Rh_2_ affects the dimerization of TLR_4_/MD-2 is not clear. The molecular docking study predicted that G-Rh_2_ would bind to the hydrophobic pocket of MD-2, which led to G-Rh_2_ occupying the position of LPS in the hydrophobic pocket of MD-2, resulting in the inability of TLR_4_ to dimerize. It has been demonstrated that amino acid residues 119–132 of MD-2 play a key role in the recognition and binding of LPS, while amino acids 46~50, 79~83 and 90~105 are key binding sites of MD-2 and TLR_4_ [3]. G-Rh_2_ interacts with MD-2 amino acids PHE119 and PHE121 that bind to important sites of LPS, blocking the binding of LPS to MD-2. In addition, G-Rh_2_ can block the binding of TLR_4_ to MD-2 ILE46 and VAL48 amino acids, blocking the formation of a complex between TLR_4_ and MD-2.

It was found that G-Rh_2_ bound to TLR_4_/MD-2 dose-dependently in SPR studies, and the binding interaction occurred in a “fast up and fast down” manner, indicating that G-Rh_2_ specifically bound to TLR_4_/MD-2 with a strong affinity, KD = 71.20 µM. Shin et al. showed that the KD value for the binding of LPS to MD-2 was 2.33 μM. Although G-Rh_2_ has a lower affinity than LPS, the results of Western blot and Co-IP showed that G-Rh_2_ significantly inhibited the formation of the TLR_4_/MD-2 complex and suppressed the downstream signal transduction. Combined with the results of molecular docking, G-Rh_2_ is mated into the hydrophobic pocket of TLR_4_/MD-2. We conjectured that the binding affinity between G-Rh_2_ and MD-2 hydrophobic pockets may be higher than that of LPS, which leads to fewer LPS lipid chains falling into hydrophobic pockets; TLR_4_ then cannot form a dimerization structure, and then its downstream signal transduction is inhibited. Regarding the crystal structure of LPS/MD-2/TLR_4_, the affinity between LPS and TLR_4_/MD-2 mainly includes the interaction between five lipid chains of LPS and residues of the hydrophobic pocket of MD-2, and the hydrophobic interaction between another lipid chain and the conserved phenylalanine of TLR_4_* [3]. More important is that the two phosphate groups on lipid A bind to the TLR_4_/MD-2 complex through interactions with positively charged amino acids in TLR_4_, TLR_4_* and MD-2. A previous study has demonstrated that these two phosphate groups within the lipid A moiety exert a profound influence on the endotoxic properties of LPS. The removal of either phosphate group results in a 100-fold reduction in endotoxic activity, with the remaining monophosphoryl LPS exhibiting relatively weak stimulatory properties with regard to the innate immune response [28]. This suggests that the affinity of LPS may depend mainly on two phosphate groups, while the affinity of LPS binding to MD-2 may be low. Therefore, LPS may have a lower affinity than G-Rh_2_ when only the capacity for binding to the hydrophobic pockets of MD-2 is compared.

Recently, lipid A derivatives like lipid IVa and eritoran, which possess four fatty acid chains, have been demonstrated to selectively interact with the hydrophobic cavity of MD-2. This interaction serves to disrupt TLR_4_ dimerization, with them acting as antagonists of the TLR_4_/MD-2 complex [29]. This also supports our hypothesis that when the number of lipid chains of LPS falling into MD-2 pockets is reduced, this becomes an inhibitor of TLR_4_ signaling transduction.

These results suggest that G-Rh_2_ is targeted by preempting LPS and TLR_4_/MD-2, and that G-Rh_2_ targeting TLR_4_/MD-2 blocks the binding of LPS to TLR_4_/MD-2 and the dimerization of TLR_4_/MD-2, which will inevitably attenuate the LPS-induced TIR wobble and the recruitment of downstream adapters. This will ultimately inhibit the activation of the NF-κB signaling pathway.

## 4. Materials and Methods

### 4.1. Main Chemicals and Reagents

The 10 × RIPA buffer and Griess reagent nitrite measurement kit were acquired from Cell Signaling Technology Co., Ltd. (Danvers, MA, USA). Mouse TNF-α and IL-6 enzyme-linked immunosorbent assay (ELISA) kits were acquired from R&D systems Co., Ltd. (Minneapolis, MN, USA). Lipopolysaccharide (LPS) and FITC-LPS (O11:B4) were purchased from Sigma Chemical Co., Ltd. (St. Louis, MO, USA). Fetal bovine serum (FBS), Dulbecco’s Modified Eagle’s Medium (DMEM), penicillin–streptomycin solution, Dulbecco’s Phosphate Buffered Saline (DPBS), GlutaMAX™-1 and 0.25% Trypsin-EDTA, 3-(4,5-dimethylthiazol-2-yl)-2,5-diphenyltetrazolium bromide (MTT) were obtained from Gibco Co., Ltd. (Waltham, MA, USA). The BCA protein assay kit was purchased from CWBIO (Beijing, China). Primary TLR_4_, MD-2, NF-κB p65, phosphorylation-NF-κB p65 (*p*-NF-κB p65) antibody, goat anti-rabbit and anti-mouse antibody were purchased from Immunoway Biotechnology. The Co-Immunoprecipitation Kit and DID cell membrane dye were purchased from Shanghai Unionway technology Co., Ltd. (Shanghai, China). His-Flag TLR_4_/MD-2 protein was acquired from BD Co., Ltd. (Franklin Lakes, NJ, USA). G-Rh_2_ (purity of 95%) was donated by professor YongRi Jin of Jilin university.

### 4.2. Cell Culture and Viability Assay

RAW 264.7 cells were obtained from the Kunming Cell Bank of Chinese Academy of Sciences and cultured in DMEM, which was supplemented with 10% FBS, 100 U/mL penicillin and 100 U/mL streptomycin at 37 °C in a humidified incubator with 5% CO_2_ [30]. The MTT assay was used for measurement of cell viability, as described by Yan et al. [31]. Cells were distributed in 96-well plates at a density of 5 × 10^5^ cells/mL and incubated for 12 h. G-Rh_2_ was prepared at 6 concentrations of G-Rh_2_ (0, 5, 10, 20, 40, 80 μg/mL) to be added to the well (nine wells were repeated for each dose group) for 24 h. Next, an MTT assay was performed in accordance with the original method. The absorbance of each well was measured at 490 nm using a microplate reader (Multiskan GO Thermo Scientiific, Waltham, MA, USA).

### 4.3. Detection of Pro-Inflammatory Factors

The dosage of G-Rh_2_ was determined by the MTT assay and a previous report [32]. In order to assay the production of TNF-α, IL-6 and NO, the supernatant of RAW 264.7 cells was collected after they were co-treated with G-Rh_2_ (7.5, 15 and 30 μg/mL) or quercetin (10 μg/mL) in conjunction with LPS (1 μg/mL) for a period of 12 h [33]. TNF-α and IL-6 levels were quantified using ELISA kits, following the manufacturer’s instructions. NO production was determined using a Griess reagent nitrite measurement kit.

### 4.4. Flow Cytometric Analysis of FITC-LPS Binding

The RAW 264.7 cells (1 × 10^6^ cells/mL) were seeded into a 6-well plate for 6 h, then incubated with FITC-LPS (5 μg/mL) with or without G-Rh_2_ (7.5, 15, 30 μg/mL) for the same time. Finally, cells in each well were collected and centrifuged at 1000 rpm for 3 min. The final cells were suspended with PBS, and the bound FITC-LPS was examined by flow cytometry (Beckman, Brea, CA, USA) according to reference [34].

### 4.5. Determination of Membrane Localization of FITC-LPS

The localization of FITC-LPS on the cell membrane was determined with reference to Hua et al. [35]. In short, RAW 264.7 cells (1 × 10^4^ cells/mL) were seeded in 6-well plates with cover slides placed in advance for 6 h, then incubated with FITC-LPS (5 μg/mL) with or without G-Rh_2_ (15 μg/mL) for the same time. Subsequently, the supernatant was removed and the cells were rinsed twice with PBS and stained with 0.2 μM DID. In the end, the cells were fixed in 4% PFA, and the localization of FITC-LPS on the cell membrane was observed with a confocal laser scanning microscope (CLSM) (ZEISS LSM900, excitation 488 nm and 507 nm, Oberkochen, Germany).

### 4.6. Western Blot Assay

A Western blot assay was carried out as previously described [31]. Briefly speaking, RAW 264.7 cells (6 × 10^5^ cells/mL) were seeded in 6-well plates for 12 h. Then, the cells were incubated with G-Rh_2_ and LPS for the same time as in the previous experiment. The total proteins were lysed, collected, determined, separated and transferred to PVDF membranes as previously described [31]. Next, the membranes were blocked with 5% nonfat milk and incubated with primary antibodies (TLR_4_, MD-2, NF-κB p65, *p*-NF-κB p65) and secondary antibodies. Finally, the membranes were detected by enhanced chemiluminescence. The protein levels were normalized against the included β-actin standards and subsequently analyzed using ImageJ software (Version 1.54j) (available at https://imagej.net/, accessed on 24 February 2023).

### 4.7. Co-Immunoprecipitation (Co-IP)

The investigation of TLR_4_/MD-2 complex formation was carried out as previously described [36]. Briefly, RAW 264.7 cells (6 × 10^5^ cells/mL) were seeded into a 6-well plate for 6 h, then incubated with G-Rh_2_ (15 μg/mL) in the presence or absence of LPS (1 μg/mL) for 12 h. Subsequently, the cells were lysed with buffer containing protease and phosohatase inhibitors. Cell extract was incubated with adequate amounts of anti-MD-2 antibody at 4 °C overnight, and then precipitated with protein A/G magnetic beads under the same conditions. After boiling, the released protein was detected by immunoblotting using anti-TLR_4_ antibody. The experimental procedure was consistent with the Western blot assay as before.

### 4.8. Molecular Docking Study

Molecular docking of G-Rh_2_ with TLR_4_/MD-2 was conducted with reference to Yao et al. [37] and performed in Autodock Vina software (version 1.1.2). Firstly, the three-dimensional structures of the compound G-Rh_2_ were downloaded from the PubChem database (https://pubchem.ncbi.nlm.nih.gov, accessed on 9 November 2022). Next, the crystal structures of TLR_4_/MD-2 (PDB ID: 2Z65) were downloaded from the PDB database (https://www.rcsb.org/, accessed on 9 November 2022). Then, we imported them into AutoDockTools software (version 1.5.6) to remove water molecules, add nonpolar hydrogens and calculate the Gasteiger charges of the structures, and saved them as PDBQT files. Lastly, the docking procedure was undertaken for the purpose of analyzing the results using both PyMOL (version 2.5) and AutoDock Vina software.

### 4.9. SPR Analysis

A Biacore T200 Biomolecular Interaction Analysis system (Company, Shanghai, China) and Series Sensor Chip CM5 (10246576) were used to determine the binding affinity of G-Rh_2_ to recombinant human TLR_4_/MD-2. SPR analysis was carried out as previously described [38]. Briefly, the TLR_4_/MD-2 protein (in acetate acid buffer pH 5.0) was loaded onto the sensors, and then an EDC and NHS mixture was added for activation of the chip. Different concentrations of G-Rh_2_ (200, 100, 50, 25, 12.5, 6.25, 3.125, 1.562 and 0 μM) were prepared with a running buffer (PBS, 0.2% Tween-20, 5% DMSO, pH 7.4). The sensor and sample plate were positioned within the instrument. Following the manufacturer’s guidelines, interactions were assessed at a flow rate of 50 μL/min, comprising an association phase of 60 s and a dissociation phase of 60 s. The collected data were analyzed using BIAcore T200 Evaluation software (version 3.2.1). Binding kinetic parameters, including KD values, were determined through global fitting of the kinetic data obtained from different concentrations of Blumeatin, employing a 1:1 Langmuir binding model.

### 4.10. Statistical Analysis

Statistical analysis was conducted using SPSS 17.0 (SPSS Inc., Chicago, IL, USA). The results are reported as mean ± standard deviation (SD) and were evaluated through one-way analysis of variance (ANOVA). *p* < 0.05 was deemed indicative of statistical significance.

## 5. Conclusions

In summary, our results showed G-Rh_2_ possessed a good anti-inflammatory effect and G-Rh_2_ could block the binding of LPS with the membrane of RAW 264.7 cells through direct interaction with TLR_4_ and MD-2 proteins, leading to the disruption of the dimerization of TLR_4_ and MD-2, followed by suppression of the TLR_4_/MD-2 mediated downstream NF-κB signaling pathway to exert anti-inflammatory effects. Our findings indicate that G-Rh_2_ is a novel TLR_4_/MD2 inhibitor and could be a potential therapeutic candidate for inflammation.

## Data Availability

All material and data are stored at Guizhou University, College of Pharmacy, Guiyang, People’s Republic of China, and may be shared upon request directed to the corresponding author.

## Abbreviations

G-Rh2: ginsenoside Rh2; DEX: dexamethasone; LPS: lipopolysaccharide; FBS: fetal bovine serum; DMEM: Dulbecco’s Modified Eagle’s Medium; MTT: 3-(4,5-dimethylthiazol-2-yl)-2,5-diphenyltetrazoliumbromide; TLRs: toll-like receptors; MD-2: myeloid differentiation factor 2; NO: nitric oxide; DPBS: Dulbecco’s Phosphate Buffered Saline; ELISA: enzyme-linked immunosorbent assay; Co-IP: co-immunoprecipitation; SPR: surface plasmon resonance; SD: standard deviation; CLSM: confocal laser scanning microscope.
